# Novel Insights into Floral Thermogenesis: In Vivo Analyses of Mitochondrial Dynamics in *Nelumbo nucifera* Flowers

**DOI:** 10.3390/ijms231911950

**Published:** 2022-10-08

**Authors:** Ruoyi Li, Jing Li, Siqin Wang, Ruohan Wang

**Affiliations:** 1 National Engineering Research Center of Tree Breeding and Ecological Restoration, Key Laboratory for Genetics and Breeding of Forest Trees and Ornamental Plants, Ministry of Education, College of Biological Sciences and Biotechnology, Beijing Forestry University, Beijing 100083, China; 2 Protein Center, Tsinghua University, Beijing 100084, China

**Keywords:** *Nelumbo nucifera*, thermogenesis, mitochondria, ROS, AOX

## Abstract

Animal-like thermogenic (TM) activities in flowers have been reported in several families of seed plants. While an association of mitochondria with floral thermogenesis has been described, how mitochondrial dynamics are involved in the regulation of floral thermogenesis is unclear. In this study, the morphological and functional dynamics of mitochondria in vivo were assessed in *Nelumbo nucifera* Gaertn. flowers during floral thermogenesis. The results showed that mitochondrial biogenesis increased considerably in *N. nucifera* flowers during thermogenesis, accompanied by notable morphological changes in the mitochondria, including long elliptical, rod-shaped, and dumbbell-shaped morphologies, as well as increased mitochondrial reactive oxygen species (ROS) levels in TM cells. An increase in the expression of alternative oxidase (AOX) during the thermogenesis of *N. nucifera* flowers was also observed. These observations suggested the rapid change in mitochondrial morphology and increased density during thermogenesis implied activation of mitochondrial fission, which combined with elevated levels of mitochondrial ROS trigger a substantial increase in AOX within the respiratory pathway of TM *N. nucifera*.

## 1. Introduction

Animal-like thermogenesis was first reported in Arum by Lamarck (1778), but is now widely recognized in many plant species. Flowers are the most notable heat-producing organ in plants, as demonstrated in 14 plant families [1,2]. Ecologically significant effects of floral thermogenesis includes an increase in floral temperature [3,4,5], which generates heat rewards for pollinating insects [5,6,7]. Wang et al. [8] experimentally showed that floral thermogenesis in *Magnolia sprengeri* promotes pollinator visitation by enhancing odor emission. Liu et al. [9] reported that the heat produced by the thermogenic (TM) flowers of *M. denudata* is retained through temporary closure of the flowers, thus benefiting anther development, pollen release, and subsequent fruit development. Recently, thermogenesis was shown to be well synchronized with insect and flower development, thereby ensuring successful reproduction [10]. However, despite the multiple biological functions of floral thermogenesis, the underlying regulatory mechanisms remain elusive.

Floral thermogenesis is accompanied by a substantial increase in respiratory activity, but is not a by-product of respiration [11,12]. The discovery of cyanide-insensitive cellular respiration in a TM plant led to recognition of a potential link between the alternative respiration pathway and regulation of floral thermogenesis [13,14]. Since the identification and characterization of the alternative oxidase (AOX) genes, much has been learned about the alternative respiration pathway [15,16,17]. For example, the terminal oxidase AOX of the mitochondrial electron transport chain was shown to act as a “heater” in plants [18,19]. AOX mediates an alternative respiration pathway that branches off from the cytochrome pathway at the ubiquinol pool, in which ATP production is diminished due to reduced proton pumping along the electron transport chain. The resulting drop in free energy between ubiquinol and oxygen is “wasted” as heat [20,21,22].

Uncoupling proteins (UCPs), first identified in brown fat mitochondria of animals [23], are another major player in cellular thermogenesis. UCPs are located on the mitochondrial membrane, where they dissipate the electrochemical proton gradient across the inner mitochondrial membrane as heat, which in animals is referred to as non-shivering thermogenesis [24,25]. Homologues of animal UCPs have been discovered in plants, and their expression is increased in response to cold [26,27,28]. Recently, UCPs were found to be specifically expressed in the TM tissues of some plants at both the mRNA and protein levels, which suggested involvement of UCPs in floral thermogenesis [22,29,30].

Regardless of the regulatory mechanism underlying floral thermogenesis, the key regulators are all located in the mitochondria. Mitochondria are at the center of cellular energy metabolism, and both their morphology and biogenesis are remarkably dynamic during biological processes such as cell differentiation and division [31], root development [32], and stress responses [33]. More mitochondria are present in TM flowers, such as those of skunk cabbage, than in non-thermogenic (NTM) flowers [34]. While these studies suggest an important role for mitochondria in floral thermogenesis, the response of mitochondrial dynamics to floral thermogenesis is unknown. In this study, thermogenesis was monitored in lotus (*Nelumbo nucifera*) flowers by high-resolution macro-focusing infrared imaging, which identified the receptacle as the major TM tissue. Our analyses of the morphological and physiological dynamics of mitochondria, and of the expression of AOX and UCPs in the TM tissues of *N. nucifera*, provide insight into the mechanisms underlying floral thermogenesis.

## 2. Results

### 2.1. Biogenesis of Mitochondria during Floral Thermogenesis

**Figure 1:**
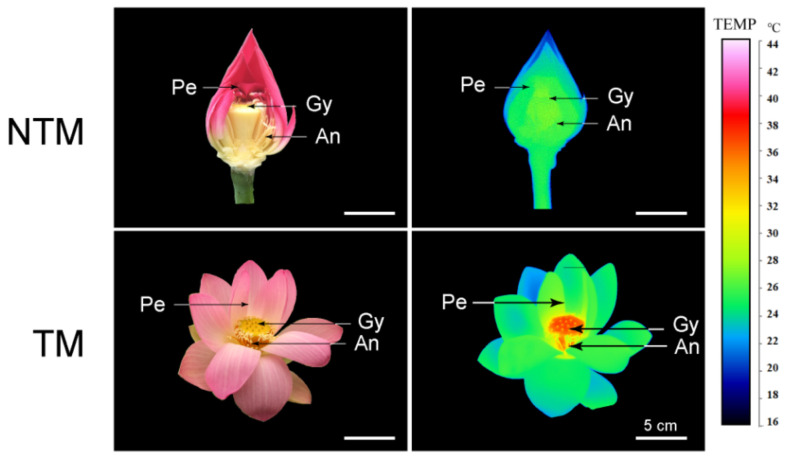
Photographs (**left**) and infrared thermal images (**right**) of *N. nucifera* flowers at the non-thermogenic (NTM; bud) and thermogenic (TM; female) stages. Temperatures in the infrared images are color coded, with red and green indicating higher and lower temperatures, respectively, in the receptacle. Pe, petal, Re, receptacle, An, anthers. Scale bars, 5 cm.

**Figure 2:**
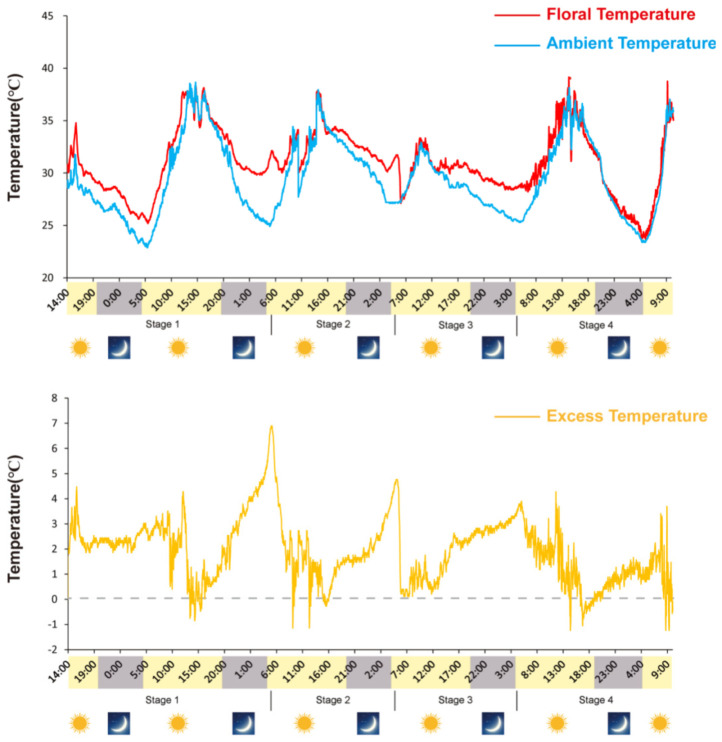
Variations in ambient (Ta) and flower (Ts) temperatures during *N. nucifera* flowering. Floral thermogenesis occurred during stage 1 (swollen stage), stage 2 (female stage), stage 3 (male stage) and stage 4 (fallen stage) but not during the non-thermogenic bud stage, the thermogenic peak (33.8 °C) was reached during stage 2; the ambient temperature was 22.3 °C.

Receptacles of *N. nucifera* showed remarkable thermogenesis during the female stage (Figure 1), as their temperature reached 33.8 °C when the ambient temperature was 22.3 °C (Figure 2). Thermogenesis in *N. nucifera* began during bud blooming, with TM peaks corresponding to the apparent receptivity of the receptacle during the female phase (Figure 2, stage 2) and the incipient shedding of pollen during the male phase (Figure 2, stage 3). As shown in Figure 2, thermogenesis in *N. nucifera* continued until the end of pollen dissemination. The sharp TM peak that occurred during the female phase was regarded as a typical TM stage.

**Figure 3:**
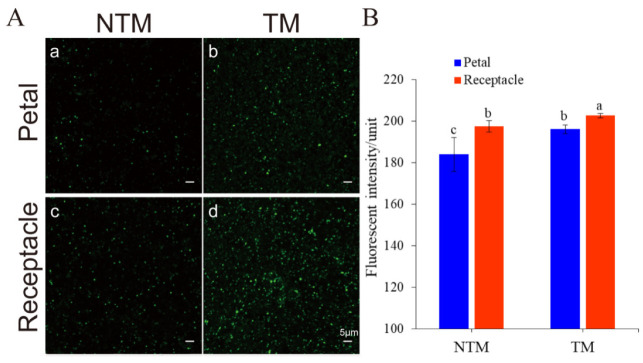
Mitochondrial dynamics in the receptacle and petals during the NTM and TM stages, as determined using MitoTracker Green (MTG). (**A**) Mitochondria labeled with MTG: Mitochondria in the petals during the NTM (**a**) and TM (**b**) stages. Mitochondria in the receptacle during the NTM (**c**) and TM (**d**) stages. (**B**) Comparison of mean fluorescence intensity in the receptacle and petals during the NTM and TM stages. The mean mitochondrial intensity in the petal during the TM (female) stage and NTM (bud) stage were 196.06 ± 2.04 and 184.00 ± 8.13, respectively. Additionally, the receptacle during the TM (female) stage and NTM (bud) stage were 202.69 ± 1.06 and 197.41 ± 2.83, respectively. The different lowercase letters indicate a significant difference in fluorescence intensity of mitochondria (**B**) between the floral stages according to the *t*-test. Data were analyzed from 10 flowers of TM and NTM stage, 3 sections (thermogenic vs. non-thermogenic) of each flower, 6 view fields for observation (88.56 μm^2^ per field). Data in the bars are the mean ± standard error and compared using the t test analysis. *p* < 0.01. Scale bars, 5 µm.

The association between mitochondrial activity and thermogenesis was examined using LSCM, to estimate the fluorescence intensity of mitochondria in receptacles and petals during the TM and NTM stages. As shown in Figure 3, MTG-labeled mitochondria in receptacle cells were highly fluorescent during the TM stage. Changes in mitochondrial density in the flowers during the TM and NTM stages were then explored by measuring the mean fluorescence intensity of mitochondria in flower tissues. The mean mitochondrial intensity of the receptacle during the TM (female) stage was 202.69 ± 1.06 (*n* = 10 flowers, three tissue sections per sample (receptacles and petals), six view fields for observation, 88.56 μm^2^ per field), which was higher than in either the NTM (bud) stage and other tissues (Figure 3B). The increase in mitochondrial fluorescence intensity indicated an increase in mitochondrial content, and thus the activation of mitochondrial biogenesis; this may have accounted for the high-level dynamics during energy metabolism.

### 2.2. Changes in Mitochondrial Morphology during Floral Thermogenesis

**Figure 4:**
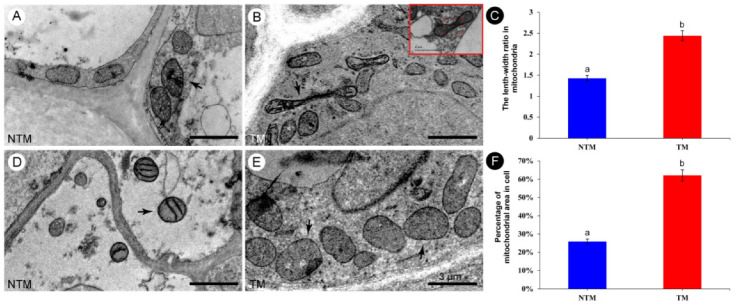
Morphology of mitochondria in the receptacle during the NTM and TM stages. (**A**,**D**) Morphology of mitochondria in the receptacle during the NTM (bud) stage. Most of the mitochondria are round or elliptical. (**B**,**E**) Morphology of mitochondria in the receptacle during the TM (female) stage. Most of the mitochondria are rod-shaped, long, elliptical, or dumbbell-shaped, and some are obviously elongated. Red rectangular box shows that the mitochondrion was dividing and was metabolically active. (**C**) Comparison of the mean length–width ratios of receptacle mitochondria during the NTM and TM stages. (**F**) Percentage of mitochondria to cell area in receptacle during NTM stage and TM stage. The different lowercase letters indicate a significant difference in the length–width ratio of mitochondria (**C**) and the mitochondrial coverage of cell (**F**) between the floral stages according to the *t*-test. Data in the bars are the mean ± standard error, 74–91 mitochondria per tissue section in (**C**) and 13–20 cells per tissue section of NTM and TM stage in (**F**). *p* < 0.001. Scale bars, 3 µm.

Mitochondrial biogenesis at the TM and NTM stages was evaluated using high-resolution TEM photographs, based on mitochondrial morphology. Since the mitochondrial fluorescence intensity was higher in the receptacle than petals, mitochondria in the former were examined (Figure 4). Receptacle mitochondria during the TM stage were elongated and tube-like, with both bends and folds (Figure 4B). During floral thermogenesis, the length–width ratio of mitochondria of receptacle reached to 2.44 ± 0.19 (Figure 4C), and the mitochondrial coverage of TM cells was as high as 62.09 + 0.02% (Figure 4F). By contrast, mitochondria during the NTM stage were round and ellipsoid (Figure 4A,D), with a length–width ratio of 1.43 ± 0.06 and the mitochondrial coverage of NTM cells was 25.93 ± 0.02%. The large number of mitochondria, and their distinct morphology during the TM stage, suggested involvement in respiration during energy metabolism.

### 2.3. ROS Production during Floral Thermogenesis

**Figure 5:**
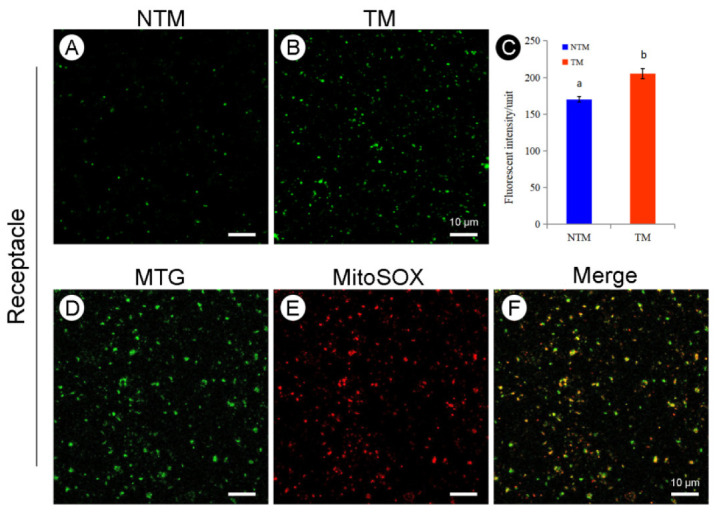
Reactive oxygen species (ROS) production and subcellular colocalization of ROS and mitochondria in the receptacle during the NTM and TM stages. (**A**) Mitochondrial ROS production in receptacle cells during the NTM (bud) stage. (**B**) Mitochondrial ROS production in receptacle cells during the TM (female) stage. (**C**) Comparison of the mean fluorescence intensity of mitochondrial ROS in receptacle cells during the NTM and TM stages. In (**C**), the different lowercase letters indicate a significant difference in fluorescence intensity of ROS between the floral stages according to the *t*-test. Data in the bars are the mean ± standard error, *n* = 10 flowers, 3 sections per sample, 6 view fields for observation in (**C**). *p* < 0.001. (**D**) Mitochondrial fluorescence intensity in the receptacle during the TM stage. (**E**) Fluorescence intensity of mitochondrial ROS in the receptacle cells during the TM stage. (**F**) Overlapping images from (**D**,**E**) show ROS accumulation in receptacle mitochondria during the TM and NTM stages. Scale bars, 10 µm.

The colocalization of mitochondria and ROS was explored by labeling the mitochondria with MTG and the ROS using Mito-SOX red (Figure 5). The results showed good coincidence of ROS and mitochondria in the receptacle during the NTM and TM stages (Figure 5F). Mito-SOX labeling of mitochondria in TM and NTM stage receptacles and petals showed significantly higher ROS accumulation in the receptacle during the TM stage than in other tissues and the NTM stage (Figure 5C). The ROS fluorescence intensity values of the receptacle during TM was obviously higher (170.26 ± 3.64) than that of NTM stages (205.49 ± 6.94, ten flowers from TM and NTM stage, respectively, three repeats per sample, six view fields for observation, 88.56 μm^2^ per field). These results implicated mitochondrial ROS in the electron transport chain during thermogenesis.

### 2.4. Abundance of AOX and UCP during Floral Thermogenesis

**Figure 6:**
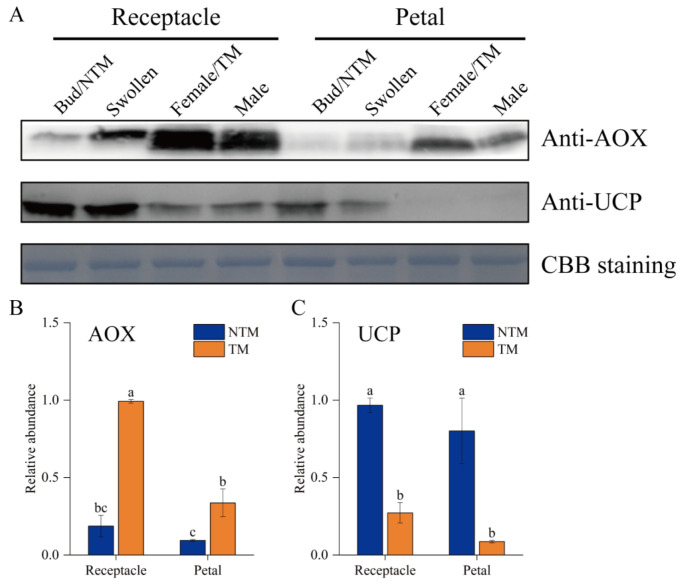
Western blot analyses of AOX and UCPs in receptacle cells during the NTM and TM stages. (**A**) Both AOX and UCPs were detected in the receptacles and petals during floral thermogenesis. Coomassie brilliant blue (CBB) staining is shown as a protein loading control. (**B**) Statistical analysis of protein abundance was based on three biological replicates for AOX and three for UCPs. Data in the bars are the mean ± standard error. AOX abundance was significantly higher during the TM than NTM stage in both the receptacles and petals. (**C**) UCP abundance were significantly higher during the NTM than TM stage in both the receptacles and petals. The different lowercase letters indicate a significant difference in protein abundance according to the *t*-test. *n* = 3 samples with three biological repeats. *p* < 0.001 versus TM receptacle group.

The potential regulatory roles of AOX and UCPs in the floral thermogenesis of *N. nucifera* were investigated by analyzing the abundance of AOX and UCPs during floral thermogenesis using Western blotting (Figure 6). Both AOX and UCPs were detected in receptacles and petals during floral thermogenesis. AOX abundance was significantly (*p* < 0.05) higher at the TM than NTM stage in both tissues (Figure 6A). Among all samples tested, AOX abundance was highest in the TM receptacle, with a 4.4-fold difference versus the NTM receptacle (Figure 6B). The UCP abundance was higher in the receptacle than in petals at the NTM stage, and the abundance of these proteins in the receptacle and petals decreased from the NTM to the TM stage (Figure 6C). This result indicated the greater regulatory importance of AOX than UCPs in the TM-stage receptacle.

## 3. Discussion

Mitochondrial activity is closely associated with physical activity in many animal and plant tissues [35,36,37]. In fluorescently stained mitochondria, higher fluorescence intensity in tissues is usually accompanied by a high mitochondrial respiration rate [38]. Our study showed that the fluorescence intensity of mitochondria was significantly higher in the receptacle than petals during both the TM and NTM stage. These results point to the receptacle as the TM tissue of *N. nucifera* flowers. The 5–10-fold increase in respiration flux during the floral TM stage [29,39,40] indicated that mitochondria are active in energy metabolism during thermogenesis.

Mitochondria are dynamic organelles that constantly undergo fission and fusion, both of which are important for mitochondrial inheritance and maintenance of mitochondrial functions [41]. Our study showed that mitochondria were more dynamic, abundant, and variable in form in TM than NTM cells. The number of mitochondria changes quickly in response to physiological activity [32,37,42,43]. In tobacco BY-2 cells, the mitochondrial density was 50% higher during exponential than stationary growth [44]. A previous study of TM brown adipose tissue (BAT) also showed that the number of mitochondria was related to energy metabolism and thermogenesis [45]. In this study, the receptacle tissues of *N. nucifera* contained more mitochondria at the TM than NTM stage. An increase in mitochondrial density at the TM stage was also reported in *Symplocarpus renifolius* [30,34]. Elongated, tube-like mitochondria were seen in TEM images of the TM stage of *N. nucifera*. A previous study showed that tube-like mitochondria undergo fission to produce two daughter mitochondria [46]. Additionally, in the TM BAT of animals, mitochondria are large and rich in cristae, while in NTM white adipose tissue they are small, with randomly oriented cristae [45]. Since the NTM stage is separated from the TM stage by only 8 h [47], the rapid change in mitochondrial density during florescence indicated that mitochondria underwent fission during this interval. Recent studies showed that mitochondrial elongation enhances cell survival, by increasing respiration to support physical activity [48], and that the transient and rapid morphological adaptations of mitochondria are crucial for many cellular processes, such as the cell cycle, immunity, apoptosis, and mitochondrial quality control [49]. In addition, a comparative analysis of TM skunk cabbage with NTM potato and cauliflower showed increased mitochondrial and metabolic activity in skunk cabbage [30]. Together, these results indicate a more active mitochondrial metabolism in TM cells. Given the link between active mitochondria and peak thermogenesis, mitochondrial dynamics in TM cells might account for the massive heat production.

The accumulation of mitochondrial ROS is an indicator of stress and thermogenesis [50,51]. For example, an increase in ROS was found in *Arabidopsis thaliana* exposed to cadmium [52]. In leaf protoplasts, salicylic acid induced mitochondrial clustering, the generation of mitochondrial ROS, and the up-regulation of AOX [53]. An increase in ROS levels was also observed in BAT after cold exposure [54,55]. We observed colocalization of ROS and mitochondria during the TM stage, thus suggesting the involvement of ROS in the TM regulation of *N. nucifera*.

ROS accumulation can induce the expression of AOX and UCPs during thermogenesis and chilling [56,57,58]. Our study showed an abundance of ROS in TM-stage receptacles of *N. nucifera*. The inhibitory effect of AOX proteins on ROS has been widely reported [22,59,60], and increased mitochondrial ROS levels act as a signal for UCP-dependent thermogenesis [51,58,61]. Based on the increased mitochondrial ROS in this study, as well as the parallel increase in AOX expression, all of these indicated a central role for AOX in the floral thermogenesis of *N. nucifera*, via a decrease in ROS production. The negative relationship between UCP and ROS demonstrated in previous studies suggests that UCPs participate in the dissipation of mitochondrial ROS levels [57,62]. In tomato plants, chilling increased ROS production in leaves and up-regulated AOX and UCP, which in turn lowered ROS accumulation to maintain the redox balance [63]. In earlier work, we identified the target genes of thermogenesis-related microRNAs [64] and found that these genes were enriched in the functional groups “photosynthetic electron transport” and “polyprenyl transferase activity.” Polyprenyl transferase is an enzyme localized to the mitochondria in plants; it transfers polyprenyl diphosphate to a benzoquinone moiety; limits the rate of coenzyme Q (CoQ) biosynthesis, which is essential for electron transport in mitochondrial respiration and antioxidant defenses [65,66]; and may interfere with the assembly or stability of respiratory chain enzymes, resulting in unbalanced oxidative phosphorylation and thus ROS production, as well as the oxidation of lipids and proteins [67,68]. Based on the increase in mitochondrial ROS production during the TM stage seen in this study, the enrichment of genes involved in mitochondrial polyprenyl transferase activity seen in our previous study), and the link between increased mitochondrial ROS and increased expression of AOX (this study), our findings indicate that active mitochondria increase ROS generation, which triggers a substantial increase in AOX in the respiration pathway; in turn, this causes the removal of excess ROS to ensure homeostasis in TM cells during floral thermogenesis.

## 4. Materials and Methods

### 4.1. Plant Materials

Lotus (*Nelumbo nucifera* Gaertn.) flowers were collected from Sanqing pond, located on the campus of Beijing Forestry University (40°00′03″ N, 116°20′25″ E).

### 4.2. Floral Thermogenesis Measurements

Floral temperatures were measured in eight individual flowers using a multichannel data acquisition device, to determine the central temperature of the flowers at 2 min intervals. Modified infrared thermal imaging (TiX1000; Fluke, Everett, WA, USA) was used to determine the temperature distribution of the flowers. A probe connected to a multi-channel acquisition device (LR-8450; Hioki, Japan) was freely suspended in air to measure air temperatures; other probes were placed close to the flowers’ receptacle to measure floral temperature. Flower cover was used for shading treatments, thus avoiding any influence of sunlight. The thermal images were processed using Smart View (v3.14; Fluke) software. The temperature of each part of the flower at different time points was recorded, and the TM tissue was thus identified.

### 4.3. Biogenesis of Mitochondria during Floral Thermogenesis

Ten flowers were sampled at the TM (female) and NTM (bud) stages, respectively. The receptacles and petals of each flower were cut into 2–3 mm-thick slices (0.30 g) using a scalpel with three biological repeats and then stained with 200 nM MitoTracker Green (MTG, Molecular Probes, Eugene, OR, USA) in 10 mM of phosphate-buffered saline (PBS). After a 20 min incubation in MTG at 25 °C, the samples were rinsed three times with 10 mM PBS and then mounted on slides with 6 view fields for microscopy observation. An inverted laser scanning confocal microscopy (LSCM; 710; Carl Zeiss, Jena, Germany) was used for mitochondrial observations and image capture. For MTG detection, the excitation wavelength was 488 nm and the emission wavelength was 500–540 nm.

### 4.4. Morphological Dynamics of Mitochondria during Floral Thermogenesis

Petals and receptacles were collected from ten flowers at the TM and NTM stages, respectively, cut into 1-mm^3^ blocks, and fixed in 2% glutaraldehyde plus 2.5% paraformaldehyde in 100 mM PBS (pH 7.5). The fixed sample blocks were kept overnight at 4 °C, rinsed three times in 100 mM PBS, and post-fixed with 1% osmium tetroxide for 4 h at room temperature. After dehydration in a graded series of ethanol (30%, 50%, 70%, 80%, 90%, 95%, and 100%), the samples were embedded in spur resin. Ultrathin sections (70-nm-thick) were prepared using an ultramicrotome (Ultracut UCT; Leica, Wetzlar, Germany), transferred to Formvar-coated grids, stained with 4% uranyl acetate, and observed by transmission electron microscopy (TEM; JEM-1010; JEOL, Japan).

### 4.5. Abundance of Reactive Oxygen Species (ROS) during Floral Thermogenesis

The dynamics of subcellular ROS from ten flowers at the TM and NTM stages were investigated by extracting the mitochondria of receptacles and petals using the methods of Day et al. [69] and Grant et al. [59], with some modifications. Lotus tissue (0.30 g) was extracted in cold mitochondrial grinding buffer, and the extract was filtered through a 50 µm nylon net prior to centrifugation at 2000× *g* for 10 min. The supernatant was then centrifuged for 20 min at 12,000× *g*. The mitochondria thus extracted were labeled with 2.5 μM MitoSOX for 15 min. ROS production was monitored in 3 sections per sample (6 view fields per section) by confocal laser microscopy. Mitochondrial basic buffer was added to the sample and the mitochondria were observed and imaged by LSCM (710; Carl Zeiss, Jena, Germany). For MitoSOX, the excitation wavelength was 543 nm and the emission wavelength was 560~620 nm.

To capture the colocalization of ROS and mitochondria, MTG fluorescence was visualized using an excitation wavelength of 488 nm and emission wavelength of 492–540 nm, and MitoSOX fluorescence at an excitation wavelength of 543 and emission wavelength of 566–650 nm.

### 4.6. Mitochondrial Protein Expression in N. nucifera

The abundances of AOX and UCP proteins were quantified based on the expression profiles of proteins in mitochondria isolated from the receptacles and petals of *N. nucifera* at the TM and NTM stages. Mitochondria were isolated from fresh tissues as described in a previous study [35], with a minor modification. Total protein was extracted from isolated mitochondria in lysis buffer. After the lysates had been washed by centrifugation at 1300× *g* for 10 min, the supernatants were subjected to Western blot analysis to determine the relative abundances of AOX and UCP, using a 1:2000 dilution of the anti-AOX1/2 antibody (AS04054; Agrisera AB, 21 Vännäs, Sweden) and 1:500 dilution of an anti-UCP1/2 antibody (AS121850; Agrisera AB, 21 Vännäs, Sweden). After incubation with the primary antibodies, the membrane was washed three times in TBST for 10 min and incubated for 4 h in a 1:2000 dilution of secondary antibody, consisting of goat anti-rabbit HRP (Pierce, Waltham; MA; USA).

### 4.7. Data Analysis

Images obtained by LSCM were analyzed using ImageJ 1.48v (National Institutes of Health, Bethesda, MD, USA). Digital pixels were considered representative of ROS and mitochondrial fluorescence intensity. Statistical analyses were performed using SPSS Statistics (ver. 23.0; IBM Corp., Armonk, NY, USA). Data are expressed as mean ± standard error. A *t*-test is applied to comparison and determine the statistical significance. *p* < 0.05 was considered statistically significant.

## 5. Conclusions

Our study revealed an increase in mitochondrial number and activity in the TM cells of *N. nucifera* during the TM stage. The rapid change in mitochondrial morphology and increased density during thermogenesis implied activation of mitochondrial fission. The high level of mitochondrial ROS in TM cells suggested a role for the alternative pathway in decreasing ROS formation during respiratory electron transport in plant mitochondria, and the rapid metabolic activity was closely related to the AOX and UCPs pathways. Given the link between ROS accumulation and the up-regulation of AOX during the TM stage, we propose that mitochondrial ROS trigger substantial increases in AOX as part of the respiration pathway in TM *N. nucifera*. By decreasing ROS production, AOX plays a critical role in maintaining homeostasis during the floral thermogenesis of *N. nucifera*. Finally, based on the greater importance of AOX than UCPs for regulating floral thermogenesis in *N. nucifera*, our findings also suggest that regulatory roles for AOX and UCPs in floral thermogenesis cannot be inferred based on their protein expression levels alone.

## Data Availability

All relevant data are presented in this paper. Further inquiries can be directed to the corresponding author.
